# Quantitating the Transfer of the HTLV-1 p8 Protein Between T-Cells by Flow Cytometry

**DOI:** 10.3389/fmicb.2018.00400

**Published:** 2018-03-07

**Authors:** Norbert Donhauser, Stefanie Heym, Andrea K. Thoma-Kress

**Affiliations:** Institute of Clinical and Molecular Virology, Universitätsklinikum Erlangen, Friedrich-Alexander-Universität Erlangen-Nürnberg, Erlangen, Germany

**Keywords:** HTLV-1, p8, virus transmission, protein transport, flow cytometry

## Abstract

The Human T-cell leukemia virus type 1 (HTLV-1)-encoded accessory protein p8 is cleaved from the precursor protein p12 encoded by the HTLV-1 open reading frame I. Both p12 and p8 are thought to contribute to efficient viral persistence. Mechanistically, p8 induces T-cell conjugates and cellular conduits. The latter are considered to facilitate transfer of p8 to target cells and virus transmission. Transfer of p8 between p8-expressing T-cells and recipient cells has been analyzed by immunofluorescence and live imaging. However, automatic quantitation of p8-transfer between cells has not been studied yet. Here we developed a novel method allowing time saving quantitation of p8 transfer between cells by flow cytometry. After establishing a protocol for the detection of intracellular p8 by flow cytometry and validation of p8 protein expression by western blot and immunofluorescence, we set up a co-culture assay between p8-expressing donor Jurkat T-cells and recipient Jurkat T-cells that had been prestained with a well-retained live cell dye. Upon quantitating the amount of p8 positive recipient cells with regard to the percentage of p8 expressing donor cells, time course experiments confirmed that p8 is rapidly transferred between Jurkat T-cells. We found that p8 enters approximately 5% of recipient T-cells immediately upon co-culture for 5 min. Prolonged co-culture for up to 24 h revealed an increase of relative p8 transfer to approximately 23% of the recipient cells. Immunofluorescence analysis of co-culture experiments and manual quantitation of p8 expression in fluorescence images confirmed the validity of the flow cytometry based assay. Application of the new assay revealed that manipulation of actin polymerization significantly decreased p8 transfer between Jurkat T-cells suggesting an important role of actin dynamics contributing to p8 transfer. Further, transfer of p8 to co-cultured T-cells varies between different donor cell types since p8 transfer could hardly been detected in co-cultures of 293T donor cells with Jurkat acceptor cells. In summary, our novel assay allows automatic and rapid quantitation of p8 transfer to target cells and might thus contribute to a better understanding of cellular processes and dynamics regulating p8 transfer and HTLV-1 transmission.

## Introduction

Human T-cell leukemia virus type 1 (HTLV-1), a delta-retrovirus, infects ca. 5–10 million people worldwide and is the etiologic agent of adult T-cell leukemia/lymphoma (ATLL) (Poiesz et al., 1980; Yoshida et al., 1982; Gessain and Cassar, 2012). The virus is transmitted via cell-containing body fluids such as blood products, semen, and breast milk (Gross and Thoma-Kress, 2016). Upon integration into the host cell genome, HTLV-1 persists mainly in its provirus form. *In vivo*, integrated HTLV-1 is mainly detected in CD4^+^ T-cells, and to a less extent in CD8^+^ T-cells, dendritic cells (DC), or monocytes (Richardson et al., 1990; Macatonia et al., 1992; Nagai et al., 2001; Hishizawa et al., 2004; de Castro-Amarante et al., 2015; Melamed et al., 2015). Recent work shows that HTLV-1 infects hematopoietic stem cells and that these cells differentiate into diverse cell lineages (Furuta et al., 2017).

Replication of HTLV-1 occurs either by infection of new cells, or by mitotic division and clonal proliferation of infected CD4^+^ T-cells (Carpentier et al., 2015; Turpin et al., 2017). Cell-cell contacts are a prerequisite for efficient infection of CD4^+^ T-cells, while DC can be infected cell-free with viral biofilms (Alais et al., 2015). For infection of CD4^+^ T-cells, the following mechanisms have been described: On the one hand, it is proposed that the virus is transmitted at tight cell-cell contacts via the virological synapse or via cell surface transfer of viral biofilms (Igakura et al., 2003; Pais-Correia et al., 2010). On the other hand, cellular conduits induced by the accessory protein p8 seem to be important for HTLV-1 transmission (Van Prooyen et al., 2010b).

p8 is a 70 amino acid (aa) protein that is proteolytically cleaved from the precursor protein p12 encoded by the HTLV-1 open reading frame I (Fukumoto et al., 2009): First, p12 is cleaved between amino acid (aa) 9/10 to remove an ER-retention signal. Second, a cleavage between aa 29/30 results in the p8 protein (Fukumoto et al., 2009). While the precursor protein p12 localizes to the endoplasmatic reticulum (ER) and to the *cis*-Golgi apparatus (Koralnik et al., 1993; Ding et al., 2001), p8 lacks an ER-retention signal and localizes to the cytoplasm and the cell membrane (Fukumoto et al., 2007, 2009). Both p12 and p8 are thought to contribute to efficient viral persistence (Valeri et al., 2010; Pise-Masison et al., 2014). Mechanistically, p8 induces T-cell conjugates and cellular conduits. The latter are considered to facilitate transfer of p8 to target cells and virus transmission (Van Prooyen et al., 2010b). The transfer of p8 to neighboring cells is supposed to induce T-cell anergy by decreasing T-cell receptor signaling (Fukumoto et al., 2007). Together, these potential functions of p8 could favor viral persistence in an immune competent host (Van Prooyen et al., 2010a,b; Edwards et al., 2011).

Thus far, little is known about the transfer of the mobile protein p8 between cells. Although immunofluorescence analysis and live imaging revealed that p8 transfer occurs within minutes (Van Prooyen et al., 2010b), p8 transfer between cells has not been automatically and quantitatively evaluated yet. Here, we developed a novel and simple method allowing a time saving quantitation of p8 transfer between co-cultured cells by flow cytometry. Manual quantitation of p8 expression in fluorescence images confirmed the validity of the assay. Application of the newly developed protocol indicated that transfer of p8 between cells depends on proper polymerization of the actin cytoskeleton and varies between different cell types. Summed up, use of this assay may contribute to a better understanding of cellular processes and dynamics regulating p8 transfer and HTLV-1 transmission.

## Methods

### Cell Culture

CD4^+^ Jurkat T-cells (from acute lymphoblastic leukemia) were cultured in RPMI 1640 with L-glutamine (0.35 g/l; GIBCO, Life Technologies, Darmstadt, Germany) supplemented with 45% Panserin 401 (PAN-Biotech, Aidenbach, Germany), 10% fetal calf serum (FCS; Sigma-Aldrich, Darmstadt, Germany) and penicillin/streptomycin (0.12 g/l each; Sigma-Aldrich). 293T cells were cultured in DMEM (GIBCO, Life Technologies), 10% FCS, L-glutamine and penicillin/streptomycin.

### Expression Plasmids

Plasmids p8-HA (pME18S-p12I-Δ29) and the respective control pME (pME18S) were kindly provided by Genoveffa Franchini and have been described earlier (Fukumoto et al., 2009; Edwards et al., 2014). Constructs were checked for integrity by automated sequencing.

### Transient Transfections

Jurkat T-cells (1^∗^10^7^) were transiently transfected by electroporation with a total amount of 100 μg of plasmid DNA using the *Gene Pulser Xcell^TM^ Electroporation System* (BioRad, Munich, Germany) at 290 V and 1500 μF (exponential pulse). 293T cells were seeded at 5 × 10^5^ cells per six-well. One day later, cells were transfected using *GeneJuice^®^ Transfection Reagent* (Merck Millipore, Darmstadt, Germany) according to the manufacturer’s protocol using a total amount of 2 μg DNA.

### Western Blot

At day 2 post transfection, 293T or Jurkat T-cells were washed in phosphate-buffered saline (PBS without Ca^2+^ and Mg^2+^) and lyzed in 150 mM NaCl, 10 mM Tris/HCl (pH 7.0), 10 mM ethylene-diamine tetra-acetic acid (EDTA), 1% Triton X-100, 2 mM dithiothreitol (DTT) supplemented with the protease inhibitors leupeptin, aprotinin (20 μg/ml each) and 1 mM phenyl-methylsulfonyl fluoride (PMSF; 1 mM) as described earlier (Mohr et al., 2014). Briefly, after repeated freeze-and-thaw cycles in liquid nitrogen, lysates were centrifuged at 14.000 rpm (15 min, 4°C), and supernatants containing cellular proteins were denatured in sodium dodecyl sulfate (SDS) loading dye [10 mM Tris/HCl (pH 6.8), 10% glycerine, 2% SDS, 0.1% bromphenole blue, 5% β-mercaptoethanol] for 10 min at 95°C. Subsequently, samples (50 μg) were subjected to SDS-polyacrylamide gel electrophoresis (SDS-PAGE) using the *XCell SureLock^TM^ Mini-Cell Electrophoresis System* (Thermo Fisher Scientific, Waltham, MA, United States) and transferred to nitrocellulose membranes (Whatman^®^, Protran^®^, Whatman GmbH, Dassel, Germany). Membranes were probed with rat monoclonal anti-HA-Peroxidase antibodies (clone 3F10; Roche, Mannheim, Germany), mouse monoclonal antibodies anti-β-actin (ACTB; Sigma-Aldrich/Merck, Darmstadt, Germany), or anti-glyceraldehyde-3-phosphate dehydrogenase (GAPDH; Sigma Aldrich/Merck). Secondary antibodies (anti-mouse) were conjugated with horseradish peroxidase (HRP; GE Healthcare, Little Chalfont, United Kingdom) and peroxidase activity was detected by enhanced chemoluminescence (ECL) using *INTAS Advanced Fluoreszenz und ECL Imager* (INTAS Science Imaging Instruments, Göttingen, Germany).

### Flow Cytometry

To detect p8-HA expression, 293T cells or co-cultured cells were washed in PBS and fixed in 2% paraformaldehyde (PFA; 20 min, 20°C). After one washing step in wash buffer (PBS, 0.5% FCS and 2 mM EDTA), cells were permeabilized in wash buffer containing 0.5% saponin (Sigma-Aldrich/Merck) and stained in the same buffer using anti-HA-APC or the respective isotype-matched control antibody mouse IgG1-APC (both Milenty Biotech, Bergisch Gladbach, Germany; 1:40, 10 min, 20°C). After two washing steps in wash buffer containing 0.3% saponin, cells were resuspended in wash buffer and at least 3–5 × 10^5^ events were analyzed using the *BD^TM^ LSR II* or the *BD LSRFortessa^TM^* flow cytometer (Becton Dickinson GmbH, Heidelberg, Germany). Both devices were equipped with 405 and 633 nm laser. For evaluation of data, *FCS Express V3* (De Novo Software, Glendale, CA, United States) was used. In some experiments as indicated in the figure legend, cells were either stained without permeabilization in wash buffer, or cells were stained using *Inside Stain kit* (Miltenyi Biotec) according to the manufacturer’s instructions.

To evaluate the vitality of Jurkat T-cells, cells were spun down, resuspended in PBS and analyzed using the *BD^TM^ LSR II* flow cytometer. The size of the cells (FSC, *forward scatter*) was plotted against the granularity (SSC, *side scatter*) and the percentage of living cells was assessed by gating.

### Chemicals

To manipulate polymerization of the actin cytoskeleton, Jurkat T-cells transfected with p8-HA or pME (control) were pooled, respectively, at 24 h post transfection and incubated with increasing concentrations of cytochalasin D (Sigma-Aldrich/Merck; 0.5 μM, 1 μM, 2.5 μM, 5 μM), a potent inhibitor of actin polymerization, or the solvent control DMSO. Chemicals were added for 24 h and cells were incubated at 37°C. After one washing step in Jurkat T-cell culture medium (see Cell Culture), cells were used for co-culture assays (see Co-culture Assays).

### Co-culture Assay Between p8-Expressing Donor Cells and Prestained Acceptor Jurkat T-Cells

#### Prestaining of Recipient Jurkat T-Cells

The live cell dye CellTracker^TM^ Blue CMAC (CMAC; 7-amino-4-chloromethylcoumarin; Thermo Fisher Scientific, Waltham, MA, United States; 10 mM in DMSO) was diluted in serum-free medium to a 20 μM working solution. To label acceptor T-cells, 1^∗^10^6^ Jurkat T-cells per experimental condition were spun down (1200 rpm, 5 min, 20°C) and resuspended in 1 ml of prewarmed CellTracker^TM^ Blue CMAC (20 μM). After 45 min at 37°C, cells were spun down, washed in serum-free medium for three times, and directly used in co-culture assays (see Co-culture Assays).

#### Co-culture Assays

In time course experiments, 1^∗^10^6^ Jurkat donor cells that had been transfected with p8-HA or pME (control) 48 h earlier were co-cultured with equal amounts of Jurkat acceptor cells prestained with CellTracker^TM^ Blue CMAC (Jurkat-CMAC) in 1 ml of Jurkat T-cell culture medium (see Cell Culture). For the time point 0 min, individual cell populations were fixed in 2% PFA prior to mixing of the donor and the acceptor cells. Co-cultures were incubated at 37° C for different time points (5, 30, 60 min, 24 h).

In experiments using cytochalasin D to manipulate cytoskeleton dynamics, 1^∗^10^6^ transfected Jurkat T-cells pretreated with cytochalasin D or DMSO at one day post transfection for 24 h (see Chemicals) were co-cultured with equal amounts of Jurkat-CMAC in 1 ml fresh Jurkat T-cell culture medium (see Cell Culture) for another 24 h at 37°C. As a negative control for p8 transfer (time point 0 h), Jurkat donor cells transfected with p8-HA and pretreated with DMSO for 24 h were fixed in 2% PFA prior to mixing with fixed Jurkat-CMAC cells.

In co-cultures between 293T and Jurkat cells, transfected 293T cells were overlaid with equal amounts of Jurkat-CMAC acceptor cells (3^∗^10^6^) in 3 ml fresh culture medium at 37°C for either 5 min or for 24 h.

In experiments using fixed Jurkat donor cells, 24-well plates were coated with poly-L-lysine (Sigma-Aldrich) according to the manufacturer’s instructions. Thereafter, 1^∗^10^6^ transfected Jurkat T-cells were cultured in uncoated wells or they were allowed to adhere on poly-L-lysine-coated wells for 1 h (37°C). In the latter case, suspending cells were removed prior to adding equal amounts of Jurkat-CMAC for another 24 h at 37°C.

In all experiments, co-cultured cells were spun down at the end of the respective co-culture period (4°C, 1200 rpm, 5 min), washed in PBS, fixed in 2% PFA (20°C, 20 min) and subjected to intracellular staining using saponin (see Flow Cytometry).

#### Flow Cytometry and Quantitation of p8 Transfer After Flow Cytometry

After fixation and staining of co-cultured cells (see Flow Cytometry and Co-culture Assays), living cells were gated in dot plots displaying the forward scatter (FSC) plotted against the side scatter (SSC). Thereafter, CMAC-specific fluorescence was plotted against the SSC to discriminate between CMAC-negative donor cells (either Jurkat or 293T) and CMAC-positive Jurkat acceptor cells. Finally, HA-specific fluorescence was plotted against the SSC within the CMAC-negative Jurkat or 293T donor cells, representing the efficiency of transfection (E_T_). In parallel, plotting of HA-specific fluorescence against the SSC within the CMAC-positive Jurkat acceptor cells, represented the transfer of p8 (*T*_p8_) to the CMAC-positive Jurkat acceptor cells. To calculate the relative transfer of p8 [*T*_p8(relative)_] between cells, *T*_p8_ was divided by E_T_. To subtract background fluorescence of the HA-APC antibodies, gates were set according to labeling with isotype matched control antibodies and, additionally, co-cultures of cells transfected with the control plasmid pME were analyzed and considered in the evaluation. To quantitate the relative transfer of p8 *T*_p8(relative)_, a mathematic equation was developed, which is described in more detail in the results section (see p8 Is Rapidly Transferred between Jurkat T-cells)

$$T_{\text{p8}(\text{relative})}=\frac{T_{\text{p8}({\text{p8}}_{\text{t}})}-T_{\text{p8}({\text{pME}}_{\text{t}})}}{E_{\text{T}({\text{p8}}_{\text{t}})}-E_{\text{T}({\text{pME}}_{\text{t}})}}$$

*T*_p8_ shows the transfer of p8, which corresponds to the percentage of p8-HA positive cells within CMAC-positive acceptor cells (T_p8(p8_t_)_) at a given time point *t* and which was normalized on background fluorescence of the respective control cells transfected with pME (T_p8(pME_t_)_). E_T_ represents the efficiency of transfection at a given time point t and corresponds to the percentage of p8-HA positive cells within CMAC-negative donor cells (E_T(p8_t_)_), which is corrected by background fluorescence of the respective control cells transfected with pME (E_T(pME_t_)_).

#### Immunofluorescence and Confocal Laser Scanning Microscopy

At 48 h post transfection, p8-expressing donor Jurkat T-cells or control cells (Jurkat + pME) were co-cultured with acceptor Jurkat T-cells prestained with CellTracker^TM^ Blue CMAC (see Prestaining of Recipient Jurkat T-cells). At different time points post co-culture (5, 30, 60 min, 24 h), cells were spotted on poly-L-lysine-coated glass slides and were fixed with 2% PFA (60 min, 20 °C). Cells were washed three times with PBS and permeabilized with 0.2% Triton X-100 (20 min, 4°C). After three washing steps, unspecific binding was prevented by 5% FCS/1% BSA in PBS (1 h, 20°C). Cells were stained with primary antibodies rabbit anti-HA (BioLegend, Eching, Germany; 1:100, 1 h, 20°C) followed by secondary antibodies anti-rabbit AlexaFluor 647 (Life Technologies/Thermo Fisher Scientific; 1:200, 30 min, 20°C). Slides were covered with ProLong Gold antifade reagent without DAPI (Molecular Probes/ Thermo Fisher Scientific) and analyzed by confocal laser scanning microscopy. All images were acquired using a Leica TCS SP5 confocal laser scanning microscope (Leica Microsystems GmbH, Wetzlar, Germany) equipped with a 63 × 1.4 HCX PL APO CS oil immersion objective lens. Images were analyzed using LAS AF software (Leica Microsystems GmbH). The numbers of cells expressing p8 in the CMAC-negative donor and CMAC-positive acceptor Jurkat T-cells were counted in 20 optical fields per experimental condition. *T*_p8(relative)_ was calculated by normalizing the mean percentage of p8-HA positive cells within CMAC-positive acceptor cells on the mean percentage of p8-HA positive cells within CMAC-negative donor cells. In total, 6125 cells were analyzed manually.

To stain p8 in transfected Jurkat T-cells, p8-HA-expressing Jurkat T-cells or control cells (Jurkat + pME) were spotted on glass slides with marked rings (Medco, Munich, Germany), fixed with 2% PFA (60 min, 20°C) and washed with PBS for three times. The staining protocol was performed as described above, except that cells were either permeabilized with 0.2% Triton X-100 (20 min, 4°C) or they were stained without permeabilization. Upon staining, slides were covered with Vectashield^®^ Antifade Mounting Medium with DAPI (Vector Laboratories/Biozol, Eching, Germany) and analyzed by confocal laser scanning microscopy.

### Statistics

The means of at least three independently performed experiments were compared using *t*-tests as indicated in the figure legends. *P*-values were calculated using Microsoft Excel. *P* < 0.05 was considered to be significant (^∗^), *P* < 0.01 was considered as highly significant (^∗∗^).

## Results

### p8 Protein Expression Is Detectable by Flow Cytometry

**FIGURE 1:**
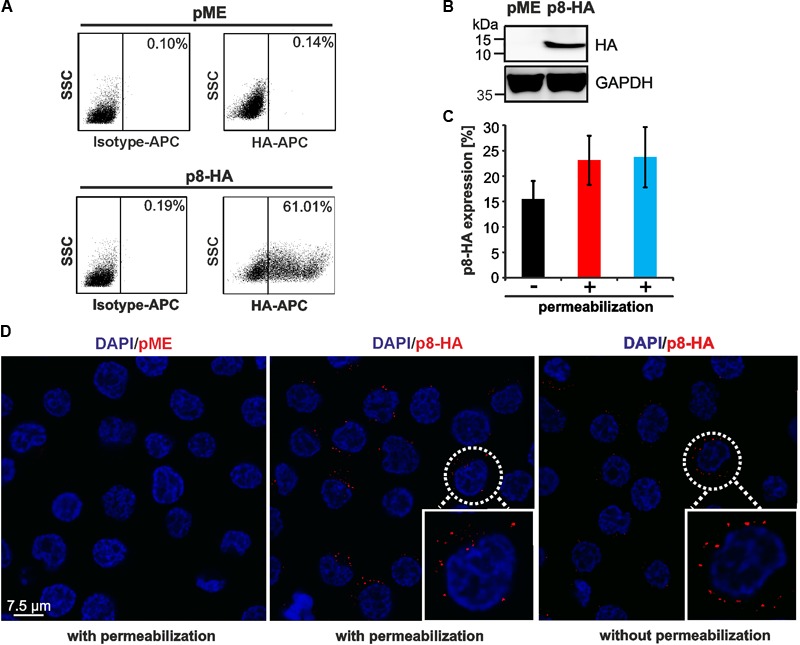
Detection of p8 by flow cytometry compared to immunofluorescence. **(A,B)** 293T cells were transfected with p8-HA expression plasmids or the control plasmid pME for 48 h. **(A)** Flow cytometry after fixation and permeabilization of cells using HA-specific, APC-labeled antibodies. Dot plots display HA-APC-specific fluorescence plotted against the side scatter (SSC). **(B)** Immunoblot of p8-HA and the housekeeping gene glyceraldehyde-3-phosphate dehydrogenase (GAPDH). **(C)** Jurkat T-cells were transfected with p8-HA expression plasmids or the control plasmid pME for 48 h. Cells were split and stained either without permeablization buffer (black bar), or upon permeabilization with 0.5% saponin (red bar) or a commercially available intracellular staining kit (blue bar). The mean percentage of p8-HA expressing cells of three experiments ± standard deviation as detected by flow cytometry is shown. **(D)** Jurkat T-cells were transfected with expression plasmids p8-HA or pME for 48 h and spotted on glass slides. Cells were stained either with or without permeabilization with HA-specific antibodies and the respective secondary antibodies. Analysis by confocal microscopy shows p8-HA (red dots) and the nuclei (DAPI). Blow up: example of a p8-HA-expressing cell.

Expression of p8 has been analyzed by western blot, immunofluorescence or live cell imaging (Fukumoto et al., 2007, 2009; Van Prooyen et al., 2010b). To also establish a protocol to detect p8 expression by flow cytometry, we transfected 293T cells with p8-HA, a p8 expression plasmid C-terminally tagged with HA, and the respective control plasmid pME. At 48 h post transfection, cells were fixed and permeabilized with saponin, followed by staining with APC-labeled anti-HA-antibodies and the respective isotype-matched control antibodies. After setting gates that considered living cells and a marker that took into account the isotype control antibodies (**Figure 1A**, upper and lower left dot plots) and the low background fluorescence of pME-transfected control cells (**Figure 1A**, upper right dot plot), we found that ca. 61% of transfected 293T cells were p8-positive (**Figure 1A**, lower right dot plot). We tested several dilutions of the anti-HA-specific antibodies (ranging from 1:10 to 1:40), however, we could detect p8 expression at every concentration with a comparable frequency and sensitivity (data not shown). In control experiments of the same samples, we could also detect robust expression of p8-HA by western blot analysis (**Figure 1B**).

Next, we asked whether p8 can also be detected in T-cells, the natural cell type of HTLV-1 infection, and whether detection of p8 expression requires permeabilization of the cells. For this purpose, Jurkat T-cells were transfected with the C-terminally tagged p8-HA plasmid or the respective control plasmid pME. At 48 h post transfection, cells were subjected to flow cytometry (**Figure 1C**) or immunofluorescence (**Figure 1D**). For flow cytometry, cells were split and stained either in absence or presence of a permeabilization buffer. In the latter case, our homemade buffer with 0.5% saponin was compared to a commercially available intracellular staining kit (kit). In all experiments, markers were considered that took into account the isotype control antibodies and the low background fluorescence of pME-transfected control cells (data not shown). Flow cytometry of non-permeabilized cells revealed that ca. 15.5% (±3.6%) of Jurkat T-cells expressed p8-HA on their surface (**Figure 1C**, black bar). Permeabilization of cells using 0.5% saponin (**Figure 1C**, red bar) or an intracellular staining kit (**Figure 1C**, blue bar) resulted in 23.1% (±4.8%) or 23.7% (±5.9%) (blue bar) of p8-HA-positive cells, respectively. First, these results show that transfection of T-cells is less efficient than transfection of 293T-cells. Second, the data suggest that the C-terminal part of p8-HA is oriented outside the cell membrane, otherwise surface staining of the HA-tagged p8 would not result in the detection of HA-positive cells. Third, these data support earlier observations that p8 is not only located intracellular, but also at the cell surface (Fukumoto et al., 2007, 2009; Edwards et al., 2014). Although staining upon permeabilization of cells resulted in a higher proportion of p8-positive cells than surface staining of p8, this difference was not statistically significant (*p* > 0.05). Next, we performed confocal microscopy of p8-transfected Jurkat T-cells either with or without permeabilization (**Figure 1D**). Imaging confirmed our results obtained by flow cytometry that p8 is detectable in both permeabilized and non-permeabilzed Jurkat T-cells. In more detail, p8 localized diffusely in dots in the cytoplasm and near the plasma membrane confirming earlier observations (Fukumoto et al., 2007, 2009; Edwards et al., 2014). Hence, to quantitate the total amount of p8 expression in cells by flow cytometry, we decided to stain p8 upon permeabilization of the cells with saponin in the next experiments. Taken together, p8 protein expression is not only detectable by immunofluorescence, but also by flow cytometry, thus, allowing automatic quantitation of p8 expression.

### A Flow Cytometry-Based Co-culture Assay Allows to Quantitate the Transfer of p8 Between Cells

**FIGURE 2:**
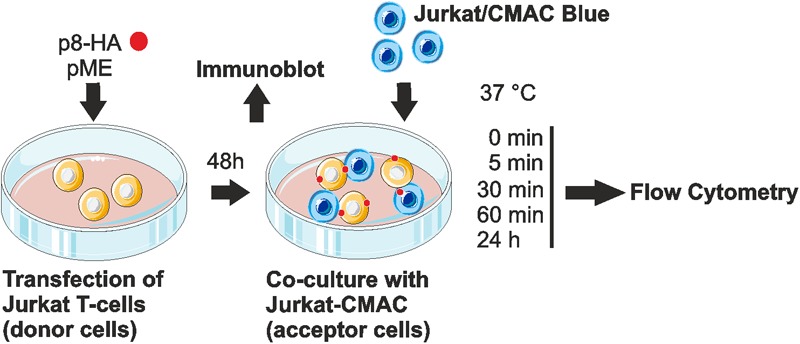
Experimental setup of the co-culture assay. Jurkat T-cells were transfected with p8-HA expression plasmids or the control plasmid pME for 48 h. Transfected p8-donor or control (pME) cells were either subjected to immunoblot analysis or co-cultured with equal amounts of Jurkat acceptor cells (1^∗^10^6^) that had been prestained with Cell Tracker Blue CMAC Dye (Jurkat-CMAC). At different time points post co-culture at 37°C (0, 5, 30, 60 min, 24 h), cells were fixed in 2% paraformaldehyde (PFA), permeabilized, stained and analyzed by flow cytometry.

Since p8 is transferred rapidly to other cells, most likely via cellular protrusions (Van Prooyen et al., 2010b), we sought of establishing an experimental setup, which allows automatic quantification of p8 transfer between T-cells by flow cytometry (**Figure 2**). Moreover, we asked whether the cell-to-cell transfer of p8 increases upon prolonged co-culture. For this purpose, Jurkat T-cells were transfected with p8-HA or the control plasmids pME. After 2 days, a part of the transfected donor cells was lyzed for a control western blot, while the remaining cells were co-cultured with equal amounts of Jurkat acceptor T-cells that were prestained with the fluorescent CellTracker^TM^ Blue CMAC (Jurkat-CMAC). Briefly, after entering living cells, CellTracker^TM^ Blue CMAC is converted into a membrane-impermeable dye, which is well retained in living cells for several generations, transferred to daughter cells, but not to neighboring cells (King et al., 2004). Thus, use of this dye allows for a proper discrimination between p8-positive, CMAC-negative donor cells and Jurkat-CMAC acceptor cells. At different time points post co-culture (0, 5, 30, 60 min, 24 h), co-cultured cells were subjected to flow cytometry.

**FIGURE 3:**
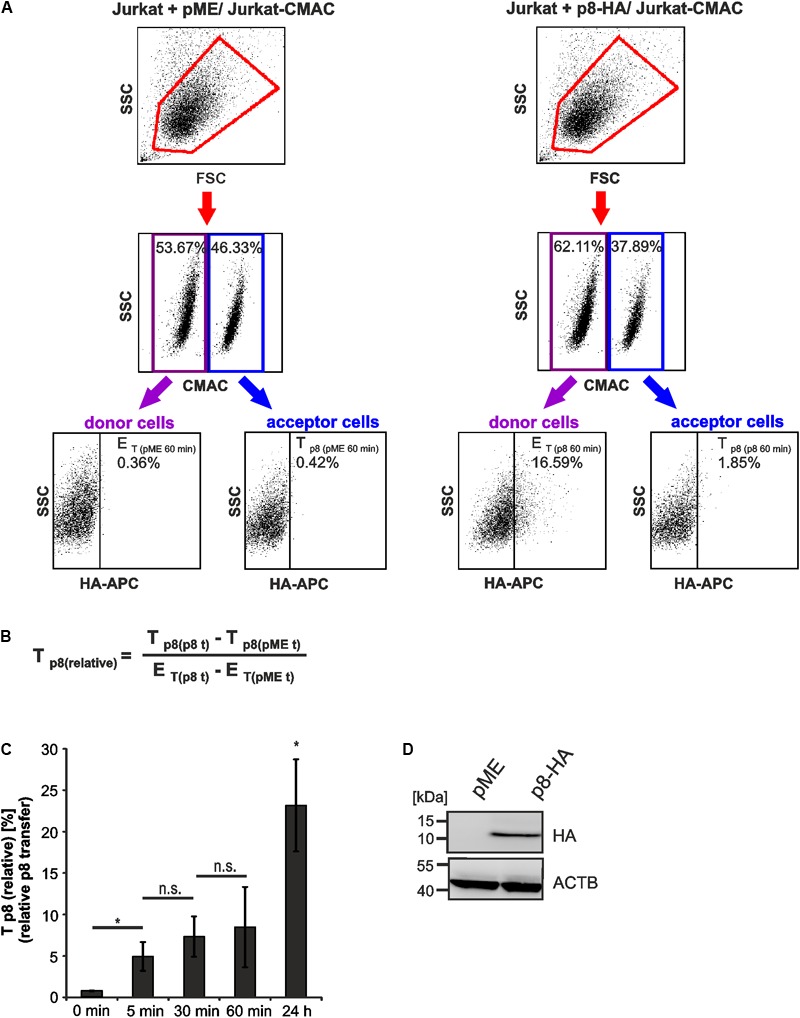
Detection of rapid p8 transfer between Jurkat T-cells by flow cytometry. Jurkat donor T-cells were transfected with p8-HA or the control plasmid pME and co-cultured with prestained acceptor T-cells Jurkat-CMAC according to the experimental setup displayed in **Figure 2**. **(A)** Flow cytometry. At 48 h post transfection, equal amounts of donor and acceptor cells (1^∗^10^6^ cells each) were either directly fixed in 2% PFA and mixed (time point: 0 min), or they were co-cultured at 37°C for 5, 30, 60 min, or 24 h before fixation. After intracellular staining using HA-specific, APC-labeled antibodies or the respective isotype-matched control antibodies, flow cytometry was performed. Representative dot plots at 60 min post co-culture are shown. First line: Dot plots display the forward scatter (FSC) plotted against the side scatter (SSC) and living cells are gated (red gate). Second line: CMAC-specific fluorescence is plotted against the SSC, which allows discrimination between CMAC-negative donor (purple gate) and CMAC-positive acceptor (blue gate) cells. Third line: HA-specific fluorescence is plotted against the SSC and numbers represent the efficiency of transfection (*E*_T_) within the CMAC-negative donor cells (displayed on the left) or the transfer of p8 (*T*_p8_) within the CMAC-positive acceptor cells (displayed on the right). **(B)** Equation to calculate the relative transfer of p8 [*T*_p8(relative)_] between cells. *T*_p8_ shows the transfer of p8, which corresponds to the percentage of p8-HA positive cells within CMAC-positive acceptor cells (T_p8(p8_t_)_) at a given time point t and which was normalized on background fluorescence of the respective control cells transfected with pME (T_p8(pME_t_)_). E_T_ represents the efficiency of transfection at a given time point t and corresponds to the percentage of p8-HA positive cells within CMAC-negative donor cells (E_T(p8_t_)_), which is corrected by background fluorescence of the respective control cells transfected with pME (E_T(pME_t_)_). **(C)** Time course analysis of *T*_p8(relative)_ as measured by flow cytometry. The means of 3–4 independent experiments ±SE are shown and were compared as indicated using a paired *t*-test. ^∗^Indicates *p* < 0.05; ^∗^ at 24 h, comparison between 24 h and all other times of measurement; n.s., not significant. **(D)** Representative immunoblot of p8-HA expression in Jurkat T-cells at 48 h post transfection. ACTB (β-Actin) served as housekeeping gene.

Co-cultured cells were analyzed by setting different gates, which are shown exemplarily for dot plots obtained at 60 min post co-culture (**Figure 3A**). First, the forward scatter (FSC) was plotted against the side scatter (SSC) and living cells were gated (**Figure 3A**, first line, red gates). Next, CMAC-specific fluorescence was plotted against the SSC (**Figure 3A**, second line) to discriminate between CMAC-negative Jurkat donor cells (purple gates) and CMAC-positive Jurkat acceptor cells (Jurkat-CMAC; blue gates). Finally, HA-specific fluorescence was plotted against the SSC to detect the efficiency of transfection (*E*_T_) within the CMAC-negative donor cells (displayed on the left) or the transfer of p8 (*T*_p8_) within the CMAC-positive acceptor cells Jurkat-CMAC (displayed on the right). Next to co-cultures of Jurkat-CMAC with p8-HA-expressing donor cells, co-cultures of Jurkat-CMAC with pME-transfected control cells were analyzed and background fluorescence of these cells was subtracted in the following evaluations. To quantitate the amount of p8 expressing recipient cells with regard to the percentage of p8 positive donor cells - the relative transfer of p8 [*T*_p8(relative)_] between cells - a mathematic equation was developed (**Figure 3B**). Exemplarily, transfection of Jurkat T-cells with p8 resulted in 16.23% (16.59% – 0.36%) of p8-HA-expressing donor cells (*E*_T_ = 16.23%; **Figure 3A**), while transfer of p8-HA was detectable in 1.43% (1.85% – 0.42%) of all Jurkat-CMAC acceptor cells (*T*_p8_ = 1.43%) at 60 min post co-culture. Normalization of transferred p8 on the transfection efficiency using the equation (**Figure 3B**) resulted in *T*_p8(relative)_) = 8.8%.

### p8 Is Rapidly Transferred Between Jurkat T-Cells

Earlier work using real time live imaging techniques had shown that p8 is transferred within minutes between cells (Van Prooyen et al., 2010b). Using our flow cytometry-based approach, we analyzed the transfer of p8 between cells over time (**Figure 3C**). Cells that were fixed before co-culture served as negative control (0 min). Compared to the negative control, *T*_p8(relative)_ significantly increased post co-culture compared to the negative control. At 5 min post co-culture, *T*_p8(relative)_ significantly increased up to 4.95% (±1.73%) while prolonged co-culture for 30 and 60 min only led to a gradual enhancement of *T*_p8(relative)_ ranging from 7.34% (±2.44%) to 8.48 % (±4.85%), which was not significantly increased when compared to the value obtained at 5 min post co-culture. This suggests that p8 is rapidly transferred within the first minutes of co-culture between cells confirming earlier findings (Van Prooyen et al., 2010b). However, prolonged co-culture of cells for 24 h resulted in a sharp and significant increase of *T*_p8(relative)_ reaching values of approximately 23.17% (±5.55%). Thus, p8 is transferred over longer periods and it accumulates over time in co-cultured cells. The expression of p8-HA was also checked by western blotting using lysates of transfected donor cells that were obtained before co-culture (**Figure 3D**). Taken together, flow cytometry allows automatic quantitation of p8 transfer between Jurkat T-cells, revealing that p8 is rapidly transferred between cells and accumulates over time.

### Comparison of Flow Cytometry and Immunofluorescence to Quantitate p8-Transfer

**FIGURE 4:**
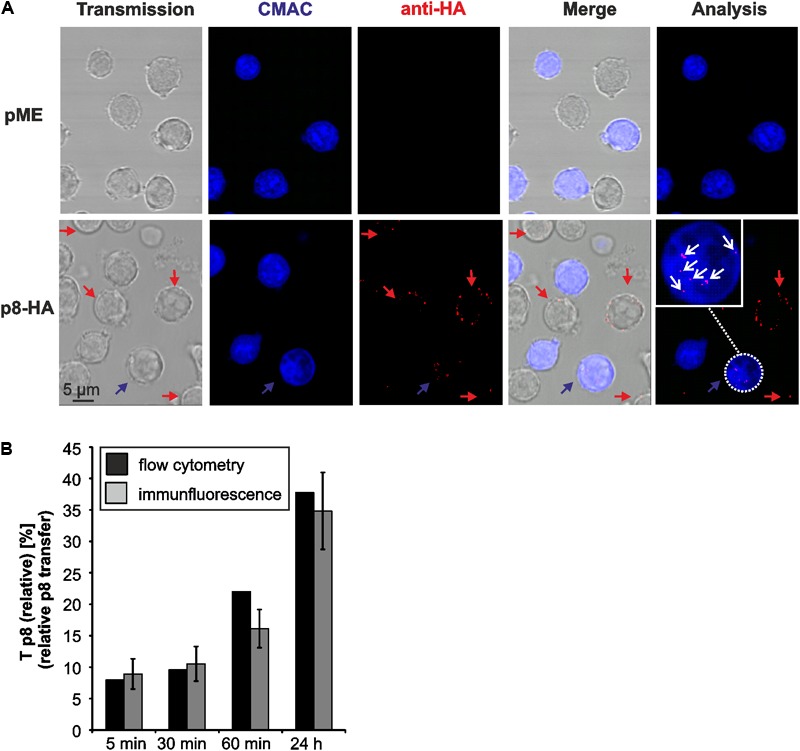
Detection of p8 transfer between Jurkat T-cells by immunofluorescence. **(A)** Jurkat T-cells were transfected with expression plasmids p8-HA or pME for 48 h and co-cultivated with equal amounts of acceptor Jurkat T-cells prestained with Cell Tracker Blue CMAC (Jurkat-CMAC) on poly-L-lysine coated glass slides for 24 h at 37°C. Thereafter, cells were permeabilized and stained with HA-specific antibodies and the respective secondary antibodies. Slides were covered with ProLong Gold antifade reagent and analyzed by confocal microscopy. A cutout of an optical field shows cells expressing p8-HA (red) within the donor Jurkat T-cells (not stained) and the acceptor Jurkat T-cells (blue). The numbers of p8-positive cells (red) within the acceptor Jurkat T-cells (blue) were counted (white circles). Red arrows: p8-positive donor cells; blue arrows: p8-positive acceptor cell; blow up: example of a p8-expressing acceptor cell; white arrows: p8-HA. **(B)** Comparison of p8 transfer between flow cytometry (black bars) and immunofluorescence (gray bars). At 48 h post transfection with p8-HA, equal amounts of p8-donor Jurkat T- cells and Jurkat-CMAC acceptor cells (1^∗^10^6^ cells each) were co-cultured at 37°C for 5, 30, 60 min or 24 h before fixation. One representative time course experiment of relative p8 transfer [*T*_p8(relative)_] as measured by flow cytometry (as shown for *n* = 4 in **Figure 3C**) is compared to the manual quantitation of relative p8 transfer within the same sample by immunofluorescence. *T*_p8(relative)_ as measured by immunofluorescence was calculated by normalizing the mean percentage of p8-HA positive cells within CMAC-positive acceptor cells on the mean percentage of p8-HA positive cells within CMAC-negative donor cells in 20 optical fields. SE, standard error.

To evaluate the validity of the flow cytometry-based assay, we performed an immunofluorescence analysis of the co-culture experiments in parallel to flow cytometry similar to the experimental setup shown earlier (**Figure 2**). Thereafter, we manually quantitated p8 expression in fluorescence images at different time points post co-culture (5, 30, 60 min, 24 h; **Figure 4**). Briefly, co-cultures of donor Jurkat T-cells transfected with expression plasmids p8-HA or pME and acceptor cells Jurkat-CMAC were spotted on poly-L-lysine coated glass slides and stained with HA-specific antibodies and the respective secondary antibodies. A cutout of a fluorescent image depicting cells co-cultured for 24 h is shown exemplarily (**Figure 4A**). Imaging revealed that the p8 protein localized diffusely in dots in the cytoplasm and near the plasma membrane in Jurkat T-cells confirming earlier observations (Fukumoto et al., 2007, 2009; Edwards et al., 2014). Moreover, we could also confirm that p8 is transferred to other cells by immunofluorescence (Van Prooyen et al., 2010b) since we detected p8 (**Figure 4A**; red dots) not only in transfected donor cells, but also in co-cultured Jurkat-CMAC acceptor cells (**Figure 4A**; blue cells; *see* blow up for details). To calculate the relative transfer of p8 [*T*_p8(relative)_] based on the imaging data, we evaluated twenty optical fields per experimental condition and counted the number of p8-positive cells (red dots, **Figure 4A**) in the CMAC-negative donor and CMAC-positive acceptor T-cells (blue; white circles; **Figure 4A**). In total, 6125 cells were analyzed. Calculation of the relative p8 transfer revealed that *T*_p8(relative)_ increased to 7.95% already at 5 min post co-culture (**Figure 4B**, gray bars). Prolonged co-culture led to a gradual increase of *T*_p8(relative)_ over time with a sharp increase at 24 h post co-culture [*T*_p8(relative)_ = 37.75%]. Comparison of the imaging data with the respective data obtained by flow cytometry (**Figure 4B**, black bars) revealed that *T*_p8(relative)_ was comparable between both methods independent of the time point of co-culture. Thus, contrary to the manual counting of immunofluorescence images, flow cytometry allows an automatic and fast quantitation of p8 protein expression in a larger number of cells.

### Transfer of p8 Between Cells Dependents on Proper Polymerization of the Cytoskeleton

**FIGURE 5:**
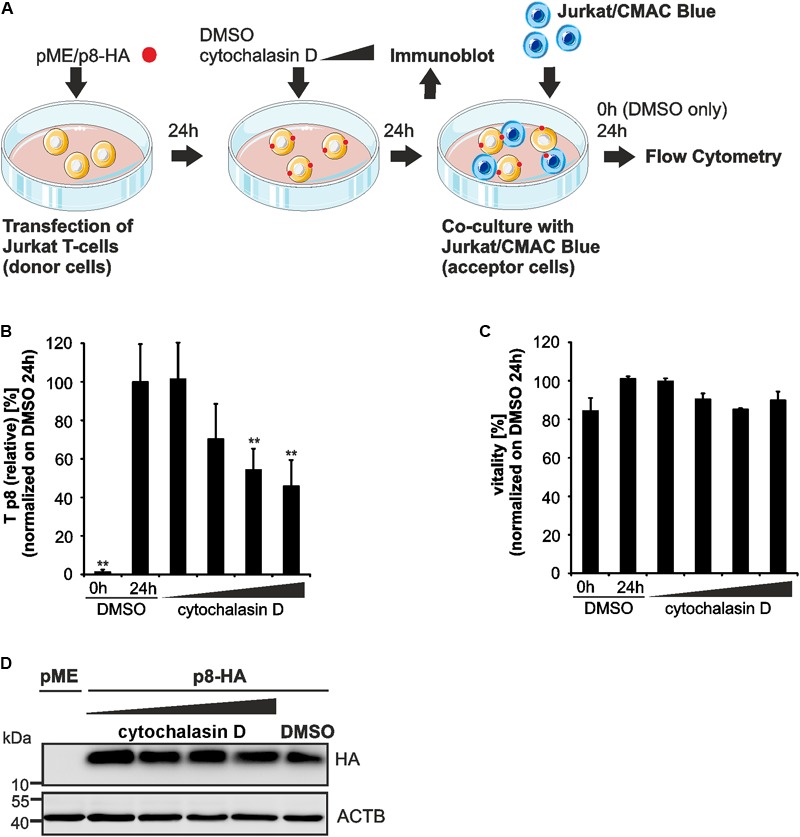
Impairment of p8 transfer between T-cells after inhibition of actin polymerization. **(A)** Experimental setup. At 24 h post transfection of Jurkat T-cells with p8-HA or pME (control) expression plasmids (100 μg each), cells were cultured with increasing concentrations of an inhibitor of actin polymerization, cytochalasin D (0.5, 1, 2.5, 5 μM), or the solvent control dimethylsulfoxide (DMSO) for 24 h. Cells were either subjected to immunoblot analysis or co-cultured in fresh medium (without chemicals) with equal amounts of prestained Jurkat acceptor cells (1^∗^10^6^ cells, labeled with Cell Tracker Blue CMAC Dye) for 24 h at 37°C and analyzed by flow cytometry. DMSO-treated donor cells were also taken at 0 h post co-culture and served as negative control for p8 transfer. **(B)** The relative transfer of p8 [*T*_p8(relative)_] was calculated as explained in **Figure 3**. Values display the means of at least four independent experiments (±SE) and were normalized on and compared to those of cells treated with DMSO and co-cultured for 24 h using an unpaired *t*-test. ^∗∗^Indicates *p* < 0.01. **(C)** Viability of Jurkat T-cells upon pretreatment with increasing amounts of cytochalasin D (0.5, 1, 2.5, 5 μM) or the solvent control DMSO for 24 h and co-culture for 0 h (DMSO) or 24 h (DMSO and all other samples) determined by forward-side scatter (FSC/SSC) analysis in flow cytometry. DMSO-treated cells (24 h co-culture) were set as 100%. The means of four independent experiments ±SE were compared to DMSO-treated cells using an unpaired *t*-test. **(D)** Representative immunoblot of p8-HA expression in Jurkat T-cells treated with the indicated inhibitors. ACTB served as housekeeping gene.

Since p8 traffics to the cell membrane and increases T-cell conjugation depending on actin polymerization (Van Prooyen et al., 2010b), we now made use of our new assay to ask whether chemical manipulation of the actin cytoskeleton affects the transfer of p8 to target cells. Therefore, Jurkat T-cells were transfected with p8-HA or pME expression plasmids (**Figure 5A**). One day later, the cells were pre-treated with increasing concentrations of cytochalasin D, an inhibitor of actin polymerization, or the solvent control DMSO for 24 h. Thereafter, a part of the transfected and pretreated donor cells was lyzed for a control western blot, while the remaining cells were co-cultured in fresh medium with equal amounts of acceptor T-cells Jurkat-CMAC for another 24 h. DMSO-treated donor cells were also taken at 0 h post co-culture and served as negative control for p8 transfer. Flow cytometry and evaluation of the results as described in **Figures 3A,B** revealed that *T*_p8(relative)_ is significantly increased between DMSO-treated donor cells and Jurkat-CMAC after 24 h of co-culture compared to the control (0 h of co-culture; **Figure 5B**). Increasing concentrations of cytochalasin D (2.5 μM, 5 μM) led to a significant and dose-dependent decline of *T*_p8(relative)_, while low concentrations did not (0.5 μM) or did only moderately (1 μM) affect *T*_p8(relative)_. To check whether cytochalasin D affected the vitality of the cells, the percentage of living cells was assessed by flow cytometry of the co-cultured cells (**Figure 5C**). Cytochalasin D did not significantly reduce the vitality of the cells in any of the concentrations tested. In parallel to flow cytometry, we also performed immunoblots, which confirmed that p8 is properly expressed under all experimental conditions (**Figure 5D**). Summed up, as an application of our new flow cytometry assay, we could now show that transfer of p8 to target cells is actin-dependent.

### Transfer of p8 Varies Between Cell Types

**FIGURE 6:**
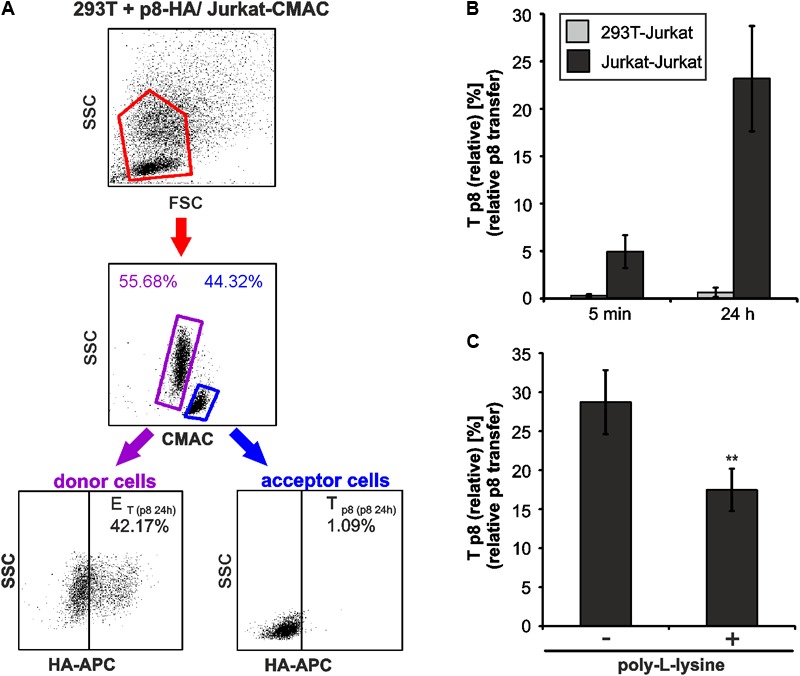
Cell type-dependence of an efficient p8 transfer. **(A,B)** 293T were transfected with p8-HA expression plasmids or the control plasmid pME for 48h. Transfected p8-donor or control (pME) cells were co-cultured with equal amounts of Jurkat acceptor cells (3^∗^10^6^) that had been prestained with Cell Tracker Blue CMAC Dye. At 5 min or at 24 h post co-culture at 37°C, cells were fixed in 2% PFA, permeabilized, stained and analyzed by flow cytometry. **(A)** Representative dot plots of 293T cells transfected with p8-HA expression plasmids after co-culture with Jurkat-CMAC acceptor cells for 24 h are shown. First line: Dot plots display the forward scatter (FSC) plotted against the side scatter (SSC) and living cells are gated (red gate). Second line: CMAC-specific fluorescence is plotted against the SSC, which allows discrimination between CMAC-negative 293T donor (purple gate) and CMAC-positive Jurkat acceptor (blue gate) cells. Third line: HA-specific fluorescence is plotted against the SSC and numbers represent the efficiency of transfection (E_T_) within the CMAC-negative 293T donor cells (displayed on the left) or the transfer of p8 (*T*_p8_) within the CMAC-positive Jurkat acceptor cells (displayed on the right). **(B)** Time course analysis of T_p8(relative)_ as measured by flow cytometry in co-cultures of 293T and Jurkat T-cells. The means of 4 independent experiments ±SE are shown and were compared as indicated using a *t*-test. For comparison, *T*_p8(relative)_ between co-cultured Jurkat T-cells as shown in **Figure 3C** is displayed. **(C)** Jurkat donor T-cells were transfected with p8-HA or the control plasmid pME. At 48 h post transfection, 1^∗^10^6^ donor cells were either fixed on poly-L-lysine coated culture plates for 1 h or they were left untreated. Thereafter, Jurkat donor T-cells were co-cultured with equal amounts of prestained acceptor T-cells Jurkat-CMAC at 37°C for 24 h and *T*_p8(relative)_ was analyzed by flow cytometry. The means of 3 independent experiments ±SE are shown and were compared as indicated using an unpaired *t*-test. ^∗∗^Indicates *p* < 0.01.

As another application for our new assay, we asked whether p8 transfer varies between different cell types. For this purpose, p8-expressing 293T donor cells and Jurkat-CMAC acceptor cells were co-cultured in analogy to the experimental setup described in **Figure 2**. At 5 min or 24 h post co-culture, cells were fixed, permeabilized and stained for flow cytometry (**Figure 6A**). Co-cultured cells were analyzed by setting gates, which are shown exemplarily for dot plots obtained at 24 h post co-culture. First, the forward scatter (FSC) was plotted against the side scatter (SSC) and living cells were gated (**Figure 6A**, first line, red gates). Next, CMAC-specific fluorescence was plotted against the SSC (**Figure 6A**, second line) to discriminate between CMAC-negative 293T donor cells (purple gates) and CMAC-positive Jurkat acceptor cells (Jurkat-CMAC; blue gates). Contrary to co-cultures in between Jurkat T-cells (**Figure 3A**), co-cultured 293T-cells and Jurkat T-cells could also be discriminated due to differences in the SSC (**Figure 6A**, second line). These data confirm that the dye CMAC is not leaky since only Jurkat T-cells (blue gate, lower SSC values) were CMAC-positive while 293T cells (purple gate, higher SSC values) remained CMAC-negative. Finally, HA-specific fluorescence was plotted against the SSC to detect *E*_T_ within the CMAC-negative 293T donor cells (displayed on the left) or *T*_p8_ within the CMAC-positive acceptor cells Jurkat-CMAC (displayed on the right). To calculate *T*_p8(relative)_, we also analyzed co-cultures of Jurkat-CMAC with pME-transfected control 293T cells (data not shown), and background fluorescence of these cells were subtracted using the mathematic equation (**Figure 3B**). Evaluation of the relative p8 transfer revealed that despite high and robust expression of p8 in 293T donor cells, p8 could only be detected at a very low frequency in co-cultured Jurkat T-cells reaching values of *T*_p8(relative)_ = 0.29% (5 min) and *T*_p8(relative)_ = 0.65% (24 h) (**Figure 6B**, gray bars). In some of the experimental replicates even none of the Jurkat acceptor T-cells was p8-positive. For comparison, *T*_p8(relative)_ within co-cultures of Jurkat T-cells was plotted (**Figure 6B**, black bars), showing that p8 transfer was much higher [*T*_p8(relative)_ = 23.17 % (24 h)]. To check whether this difference in p8-transfer between 293T and Jurkat T-cells is due to the fact that 293T cells are adherent while Jurkat are suspension cells, we allowed p8- and pME-transfected Jurkat T-cells to adhere on poly-L-lysine coated wells (1 h, 37°C) prior to addition of Jurkat-CMAC acceptor cells. After 24 h of co-culture, T_p8(relative)_ was obtained by flow cytometry (**Figure 6C**). In comparison to co-cultures between non-adherent Jurkat T-cells (without poly-L-lysine), *T*_p8(relative)_ was significantly reduced by 41.3% if donor Jurkat T-cells were fixed on poly-L-lysine-coated wells (*p* < 0.01). Despite this significant reduction of p8-transfer upon adherence of Jurkat donor cells, *T*_p8(relative)_ was still higher between fixed Jurkat T-cells and Jurkat-CMAC (**Figure 6C**; *T*_p8(relative)_ = 17.5%) than between 293T and Jurkat-CMAC (**Figure 6B**; 0.65%). Summed up, these data show that transfer of p8 depends on motility of the donor cells, and, to a greater extent, on the cell type. Thus, our novel protocol might be useful to study p8 transfer between cell types that are naturally infected with HTLV-1.

## Discussion

Quantitation of protein transport based on the evaluation of immunofluorescence images is a labor and time intensive effort. Dependent on the experimental setup, either manual or software-assisted evaluation of images is possible. Under each condition, high numbers of images with sufficient numbers of cells in several biological replicates have to be evaluated. Further, to localize and quantitate certain proteins within a cell, 3D evaluation of cells, including the time consuming analysis of Z-stacks, may be required. Certain circumstances allow the individual establishment of algorithms that may bypass the aforementioned difficulties (Wiesmann et al., 2017). To circumvent the stated problems of image evaluation, we developed a simple and fast flow cytometry-based assay to quantitate the transport of the HTLV-1-encoded protein p8 between cells. Contrary to the manual counting of immunofluorescence images, flow cytometry allows an automatic and fast quantitation of fluorescent protein expression in a large number of cells.

The viral protein p8 encoded by HTLV-1 is a mobile protein that is transferred rapidly between cells (Van Prooyen et al., 2010b). Despite low expression levels of p8 *in vivo*, it is required for establishing persistent infections (Valeri et al., 2010; Pise-Masison et al., 2014). Earlier work has shown that p8 enhances the formation of cellular conduits between T-cells, is transferred through these conduits to target T-cells and increases HTLV-1 transmission. Mechanistically, it was suggested that p8 dampens T-cell responses in target T-cells, thus facilitating HTLV-1 infection (Fukumoto et al., 2007; Van Prooyen et al., 2010a,b; Edwards et al., 2011). Thus, a better understanding of p8 transport between cells might broaden our understanding of HTLV-1 infectivity. Yet, p8 transfer between cells was analyzed by imaging techniques only (Van Prooyen et al., 2010b). However, flow cytometry based quantitation of p8-transfer provides several advantages compared to immunofluorescence-based quantitation of p8 transfer: Flow cytometry saves time, allows automatic quantitation of large number of cells, and thus, analysis of larger data sets. Further, use of CellTracker^TM^ Blue CMAC for staining of the acceptor cells provides a reliable discrimination between acceptor and donor cells since the dye is well retained in living cells (King et al., 2004), which we could also confirm in our co-culture experiments between 293T cells and Jurkat-CMAC (**Figure 6A**). This is an advantage over older dyes like Calcein-AM, which is cleaved by intracellular esterases to a fluorescent, membrane–impermeable, but gap junction–permeable form (Czyz et al., 2000). Further, the method is transferrable to be used with other cell-penetrating live cell dyes with different fluorescence. However, it is recommended to test several dilutions in co-culture experiments to ensure the dye is not leaky. Since further colors could be analyzed by flow cytometry, the method described here could be combined with methods measuring HTLV-1 virus transmission. It had been nicely shown by real-time live imaging that Gag-YFP expressed from an HTLV-1 construct and p8-mCherry are transported via protrusions to neighboring cells within minutes (Van Prooyen et al., 2010b). Combining the measurements of p8 transfer as described here and Gag transfer by flow cytometry (Gross et al., 2016) opens up the possibility to quantitatively evaluate p8’s role in virus transmission.

Despite several advantages, the flow cytometry-based assay has also some limitations since it underestimates the real transfer of p8 between cells. First, the method does not consider transfer of p8 within transfected cells since all transfected cells are counted as donor cells. Second, it is also possible that p8 is secondarily transferred within the population of CMAC-blue stained acceptor cells and from CMAC-blue stained acceptor cells back to donor cells. However, all these circumstances cannot be excluded when evaluating immunofluorescence images, too, except, using live cell imaging of individual cells. A strategy to solve this issue would be the development of a fluorescently labeled p8 protein, which changes fluorescence when entering a new cell to discriminate between donor and acceptor cells.

Making use of the method, we performed time course experiments which confirmed earlier work based on real time live cell imaging that p8 is transferred between cells within few minutes after co-culture (Van Prooyen et al., 2010b). Extending these previous findings, we also observed both by flow cytometry and imaging that p8 transfer gradually increases over time. Assuming that every donor cell is p8 positive, p8 was transported to more than every fifth co-cultured T-cell at 24 h post co-culture, suggesting that p8 is efficiently spreading and accumulating over time.

Thus far, host factors regulating the transport of p8 between cells are unknown. Earlier work has shown that p8 traffics to the cell membrane and increases T-cell conjugation depending on actin polymerization (Van Prooyen et al., 2010b). We could now extend these earlier findings and show that transfer of p8 to target cells is actin-dependent, too. In unpublished work from our group (Donhauser et al., 2018, in preparation), we found a novel interaction between p8 and a modulator of actin-filament elongation, thus supporting an important role of proper actin polymerization for efficient transport of p8 to other cells. Taken together, the flow cytometry-based assay might be useful in determining the impact of various compounds on p8’s trafficking between cells.

Yet, it is not understood whether p8 is transferred between all cell types that are HTLV-1-infected *in vivo*, or whether certain, cell-type specific host factors or co-culture conditions modulate the efficiency of p8 transfer between individual cell types. Our novel method may help to solve these questions in future studies. Data presented here clearly show that p8 transfer varies between different cell types. Despite high and robust expression of p8 in 293T donor cells, p8 could only be detected at a very low frequency in co-cultured Jurkat T-cells; in some of the experimental replicates even none of the Jurkat acceptor T-cells was p8-positive. First, host factors interacting with p8 and modulating p8 transfer may be missing in certain cell types like 293T cells. Second, the nature of cell–cell contact, the formation of cellular protrusions, and the ratio of donor and acceptor cells may be critical for p8 transfer. Third, it cannot be excluded that signals from the acceptor cell and respective receptors on the donor cell are critical for the transfer of p8, which is properly working between Jurkat T-cells but not between 293T and Jurkat T-cells. Fourth, the motility of the donor cell seems to be critical for p8-transfer since Jurkat T-cells fixed on poly-L-lysine transfer less p8 than suspension Jurkat T-cells. Lastly, it is unclear whether soluble p8 protein contributes to p8-transfer, which could be excluded by the use of filters (Chevalier et al., 2014). Hitherto, transfer of p8 has been shown by imaging techniques in co-cultures of p8-expressing Jurkat or MT-2 donor T-cells with Jurkat acceptor T-cells, or resting and activated PBMC acceptor cells (Van Prooyen et al., 2010b). Thus, further work applying our method is required to systematically and quantitatively evaluate the time course of p8 transfer in different cell types, including primary cell types like CD4^+^ T-cells, CD8^+^ T-cells, monocytes, dendritic cells and plasmacytoid dendritic cells, which are naturally infected with HTLV-1 *in vivo* (Richardson et al., 1990; Macatonia et al., 1992; Koyanagi et al., 1993; Nagai et al., 2001; Hishizawa et al., 2004; de Castro-Amarante et al., 2015; Melamed et al., 2015).

## Conclusion

Use of the novel method described in this manuscript allows automatic and rapid quantitation of p8 transfer to target cells and might thus contribute to a better understanding of cellular processes and dynamics regulating p8 transfer and HTLV-1 transmission.

## Author Contributions

ND and SH performed the experiments and analyzed the data. AT-K conceived of the study, designed the experiments, analyzed the data, and wrote the manuscript.

## Conflict of Interest Statement

The authors declare that the research was conducted in the absence of any commercial or financial relationships that could be construed as a potential conflict of interest. The handling Editor declared a past co-authorship with one of the authors AT-K.
